# Bioinformatics characterization of variants of uncertain significance in pediatric sensorineural hearing loss

**DOI:** 10.3389/fped.2024.1299341

**Published:** 2024-02-21

**Authors:** Sloane Clay, Adele Evans, Regina Zambrano, David Otohinoyi, Chindo Hicks, Fern Tsien

**Affiliations:** ^1^ Department of Genetics, Louisiana State University Health Sciences Center, New Orleans, LA, United States; ^2^ Department of Otolaryngology, Children's Hospital of New Orleans, New Orleans, LA, United States; ^3^ Department of Pediatrics, Division of Clinical Genetics, Louisiana State University Health Sciences Center and Children’s Hospital of New Orleans, New Orleans, LA, United States; ^4^ Department of Genetics, Bioinformatics and Genomics Program, Louisiana State University Health Sciences Center, New Orleans, LA, United States

**Keywords:** variants of uncertain significance, pediatric hearing loss, Usher Syndrome, *ADGRV1*, *VLGR1*, bioinformatics

## Abstract

**Introduction:**

Rapid advancements in Next Generation Sequencing (NGS) and bioinformatics tools have allowed physicians to obtain genetic testing results in a more rapid, cost-effective, and comprehensive manner than ever before. Around 50% of pediatric sensorineural hearing loss (SNHL) cases are due to a genetic etiology, thus physicians regularly utilize targeted sequencing panels that identify variants in genes related to SNHL. These panels allow for early detection of pathogenic variants which allows physicians to provide anticipatory guidance to families. Molecular testing does not always reveal a clear etiology due to the presence of multigenic variants with varying classifications, including the presence of Variants of Uncertain Significance (VUS). This study aims to perform a preliminary bioinformatics characterization of patients with variants associated with Type II Usher Syndrome in the presence of other multigenic variants. We also provide an interpretation algorithm for physicians reviewing molecular results with medical geneticists.

**Methods:**

Review of records for multigenic and/or VUS results identified several potential subjects of interest. For the purposes of this study, two *ADGRV1* compound heterozygotes met inclusion criteria. Sequencing, data processing, and variant calling (the process by which variants are identified from sequence data) was performed at Invitae (San Francisco CA). The preliminary analysis followed the recommendations outlined by the American College of Medical Genetics and Association for Molecular Pathology (ACMG-AMP) in 2015 and 2019. The present study utilizes computational analysis, predictive data, and population data as well as clinical information from chart review and publicly available information in the ClinVar database.

**Results:**

Two subjects were identified as compound heterozygotes for variants in the gene *ADGRV1*. Subject 1's variants were predicted as deleterious, while Subject 2's variants were predicted as non-deleterious. These results were based on known information of the variants from ClinVar, multiple lines of computational data, population databases, as well as the clinical presentation.

**Discussion:**

Early molecular diagnosis through NGS is ideal, as families are then able to access a wide range of resources that will ultimately support the child as their condition progresses. We recommend that physicians build strong relationships with medical geneticists and carefully review their interpretation before making recommendations to families, particularly when addressing the VUS. Reclassification efforts of VUS are supported by studies like ours that provide evidence of pathogenic or benign effects of variants.

## 1 Introduction

Pediatric sensorineural hearing loss (SNHL) affects nearly 40,000 children annually in the United States and occurs bilaterally in two-thirds of these cases. Genetic testing is an essential component of a comprehensive medical evaluation for pediatric sensorineural (SNHL) because more than half of pediatric SNHL cases have a genetic component. Pathogenic changes in genes (variants) may lead to isolated SNHL (non-syndromic) or a syndromic SNHL. Early identification of pathogenic variants contributing to syndromic SNHL allows otolaryngologists, geneticists, and other practitioners to provide anticipatory guidance as well as grant families access to medical and community resources that anticipate disease sequelae (1–5). For example, pathogenic variants linked to Usher Syndrome, inherited in an autosomal recessive manner, cause bilateral SNHL and retinitis pigmentosa (RP) (2–4). The three types of Usher Syndrome (types 1, 2, and 3) differ in their contributing genes and onset of the clinical features, the most common being Type 2 (USH2). The genes *USH1C, CDH23, PCDH15*, and *USH1G* correspond to Usher type 1*; USH2A, ADGRV1*, and *WHRN* to Usher type 2; and *CLRN1* to Usher type 3 (2–4, 6, 7). Usher Syndrome may also be inherited in digenic, biallelic, and polygenic forms as these genes interact in a protein complex (3, 4, 6, 7).

**Figure 1:**
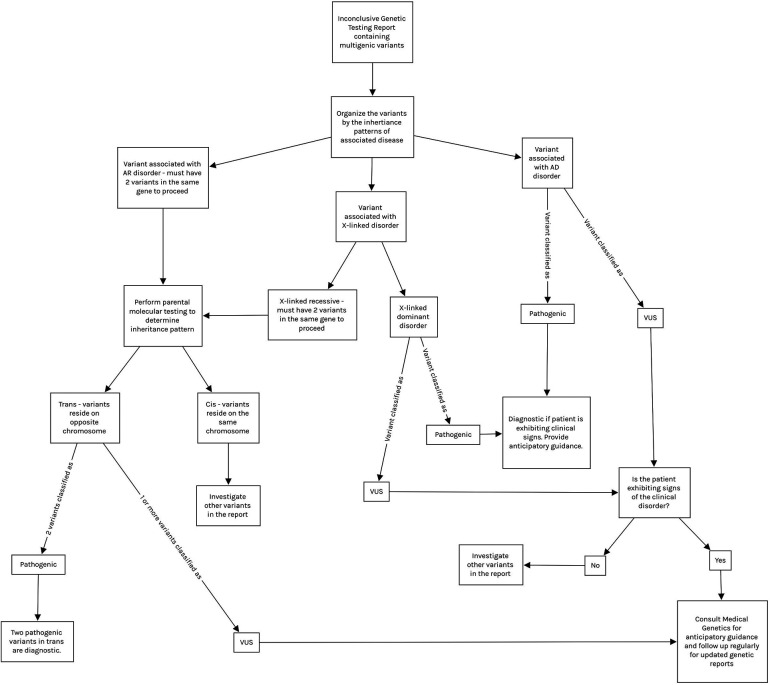
Demonstrates a proposed interpretation algorithm for physicians reviewing inconclusive molecular results and indicates when referral to medical genetics is appropriate. All interpretation of molecular results should be done with the guidance of medical genetics. Note that variants in mitochondrial genes are tested via separate panels from autosomal genes, and thus are not included in this algorithm. Mitochondrial variant testing should be considered if clinical suspicion is high. AD, autosomal dominant; AR, autosomal recessive; VUS, variant of uncertain significance.

The genetic heterogeneity of Usher Syndrome can lead to late or incorrect diagnosis, thus a thorough analysis of any genetic report containing variants in Usher genes is warranted (Figure 1) (2–4, 6). Variants for recessive diseases are often inherited in a compound heterozygous manner, meaning they inherit two distinct variants in the same gene (one from each parent) as opposed to the same variant on each chromosome (in trans). Inheritance must be confirmed with parental testing to ensure the variants reside on opposite chromosomes. Additionally, both variants must be pathogenic in order to confirm the diagnosis. “Variants of uncertain significance” (VUS), may be encountered during the diagnostic process, meaning that the effect of the variant on the phenotype is currently unknown. If the variants are classified as VUS, the syndrome may be suspected but cannot be confirmed. VUS in compound heterozygotes may be reclassified to Pathogenic, Likely Pathogenic, Likely Benign, or Benign upon further analysis and parental testing (2, 8). VUS reclassification is a dynamic process and thus families require regular follow up and should be counseled on the potential for their genetic diagnosis to change (1, 5, 8).

Rapid advancements in sequencing technologies and bioinformatic tools for genomic data analysis during the last decade have increased the utilization of next-generation sequencing (NGS) and have generated large data sets (9, 10). Furthermore, the dramatic decrease in cost and easy access to NGS assays have accelerated its use by clinicians and researchers (10). Many health care providers utilize commercially available targeted sequencing panels that test for variants in a select number of genes with known associations to hearing loss (3, 8). These variants can be studied using computational (*in-silico*) models to predict variant pathogenicity. These programs analyze information regarding the variant's position in the transcript and protein, biochemical impact, and level of evolutionary conservation to predict the effect of the variant on the gene product (8).

In this study, we examined a cohort of two pediatric subjects with bilateral SNHL who underwent genetic testing via a targeted hearing loss panel. Both patients are compound heterozygotes for variants in *ADGRV1*, a gene implicated in USH2 [bilateral SNHL at birth and retinitis pigmentosa (RP) in the second decade of life] (2–4). Our aim was to evaluate the potential pathogenicity of *ADGRV1* variants utilizing an *in-silico* bioinformatics approach to predict whether the variants could contribute to the subjects' SNHL. We propose an interpretation algorithm for physicians analyzing inconclusive genetic testing reports.

## 2 Methods

### 2.1 Characteristics of the study population

An Institutional Review Board-approved retrospective chart review was performed at this tertiary care dedicated pediatric referral center. Records were searched for sensorineural hearing loss (ICD-10 H90.3) and completed genetic testing via the Invitae Comprehensive Deafness Panel^©^ (CPT codes 81406, 81407, San Francisco CA) in patients ages 0–18 years of age. Patients with a confirmed genetic diagnosis at the time of testing were excluded.

Review of records for multigenic and/or VUS results identified several potential subjects of interest. For the purposes of this study, two *ADGRV1* compound heterozygotes met inclusion criteria (reference transcript NM_032119.3).

Subject 1 was a 2-year-old French Acadian female who was referred to the institution at 2 months of age for a failed newborn hearing screen. An Auditory Brainstem Response (ABR) revealed moderate bilateral SNHL. Her EKG was normal, and temporal bone computed tomography showed subtle left cochlear dysplasia but was otherwise interpreted as “normal”. Her prenatal and birth history were negative for non-genetic etiologies of hearing loss such as TORCH infections, prematurity, or other exposures. There was no family history of hearing or vision loss. She was treated with bilateral hearing aids. By age 2, her hearing loss had not progressed nor had she developed features of RP.

Subject 2 was a 6-year-old African American male who presented to the institution at age 4 years. His past medical history included congenital corneal endothelial dystrophy (CHED), congenital glaucoma, asthma, and SNHL beginning at age 4. During the course of this study, he was diagnosed with Harboyan Syndrome, consistent with CHED in the presence of SNHL. Both isolated CHED and Harboyan Syndrome are associated with pathogenic variants in *SLC4A11*. Subject 2 was compound heterozygous for 2 Pathogenic *SLC4A11* variants in trans. He was not diagnosed at the time of testing because he inherited a *SLC4A11* VUS that was reclassified to “Pathogenic” at a later date. He also had 2 VUS in *ADGRV1*, thus Usher Syndrome was considered in his differential diagnosis (Table 1). His hearing loss was treated with bilateral hearing aids. By age 6, his hearing loss had not progressed nor had he developed features of RP.

**Table 1:** Clinical reports for each subject. Some variant classifications were updated between the time of data collection and publication; these are noted in the “Variant classification” column. All variants were inherited in a heterozygous manner.

	Gene	Variant	Variant classification	Inheritance
Subject 1	ADGRV1	c.16172T > G(p.Leu5391Arg)	VUS	Paternal allele
	ADGRV1	c.2035C > T(p.Arg679Trp)	VUS	Maternal allele
	CACNA1D	c.6160C > T(p.Arg2054Trp)	VUS	Paternal allele; can cause AD disorder, inherited from unaffected parent
	MYO7A	c.2283G > A(silent)	VUS	Carrier
	PEX5	c.750G > C(p.Gln250His)	VUS	Carrier
	UBR1	c.3290C > T(p.Thr1097Met)	VUS	Carrier
Subject 2	SLC4A11	c.623del(p.Val208Glyfs*14)	Pathogenic	Unknown inheritance because father was unavailable for testing
	SLC4A11	c.2606 + 1 G > A	VUS reclassified as pathogenic	Maternal allele Splice donor
	ADGRV1	c.12286-10 T > C	VUS reclassified as likely benign	Intronic, unknown inheritance (not present in mother, father unavailable)
	ADGRV1	c.1283A > G(p.Asn428Ser)	VUS reclassified as likely benign	Unknown inheritance (not present in mother, father unavailable)
	COL9A2	c.683C > T (p.Pro228Leu)	VUS	
	PCHD15	c.2194G > A (p.Ala732Thr)	VUS	Carrier
	PEX5	c.610A > G (p.Lys204Glu)	VUS	Carrier
	TRIOBP	c.5150A > T (p.Gln1717Leu)	VUS	Carrier
	ZNF469	c.8095G > A (p.Asp2699Asn)	VUS	Carrier

VUS, variant of uncertain significance; AD, autosomal dominant.

### 2.2 Sources of genetic data

Sequencing, data processing, and variant calling (the process by which variants are identified from sequence data) was performed at Invitae (San Francisco CA). At the time of testing, this panel tested for variants in 203 genes related to syndromic and non-syndromic SNHL. The Invitae Comprehensive Deafness Panel^©^ (San Francisco CA) identifies exon variants as well as variants located within 10 to 20 base pairs of adjacent intronic sequence on either side of the coding exons. The data was extracted from clinical reports provided by Invitae (San Francisco CA). The patients' Invitae Comprehensive Deafness Panel clinical reports were obtained on June 10, 2022 from the institution's Genetics Clinic. For the subjects in this study, we also collected parental genetic test results on July 1, 2022. This data is summarized in Table 1 including updates received after the date of initial data collection.

### 2.3 Bioinformatics analysis of genetic variants

The preliminary analysis followed the recommendations outlined by the American College of Medical Genetics and Association for Molecular Pathology (ACMG-AMP) in 2015 and 2019. The present study utilizes computational analysis, predictive data, and population data. The ACMG recommends the use of specific standard terminology for variant classification: “Pathogenic”, “Likely Pathogenic”, “Variant of Uncertain Significance”, “Likely Benign”, and “Benign”. A variant's classification is determined by the type, strength, and category of available evidence. To address the issue of certainty, to determine whether the variant was deleterious, and to assess potential pathogenicity, the following software was used:

- PolyPhen-2 (http://genetics.bwh.harvard.edu/pph2/index.shtml) (11)—a tool that predicts possible impact of an amino acid substitution on the structure and function of a human protein.
- MutTaster (https://www.mutationtaster.org/) (12)—a tool that evaluates the pathogenic potential of DNA sequence alterations including amino acid substitutions, intronic and synonymous alterations, short insertion and/or deletions, and variants spanning intron-exon borders.
- PhyloP—a score measuring evolutionary conservation at a particular position. The scores compare the evolution of the position of interest to what would be expected from natural drift. These scores were obtained from the MutTaster report.
  a. Positive scores indicate the site is evolutionarily conserved, meaning the site is evolving slower than expected.
  b. Negative scores indicated the site is evolutionarily accelerated, meaning the site is evolving faster than expected.
- PredictSNP (https://loschmidt.chemi.muni.cz/predictsnp2/?action=format) (13)—a unified platform for the prediction of single nucleotide polymorphism effect in distinct genomic regions. This platform utilizes the five best performing tools, supplements the prediction with information from eight public databases, and generates a prediction and a confidence score for each platform utilized.
  a. CADD scores and SIFT predictions were generated using this platform.
    i. SIFT (14)—a tool that uses sequence homology to predict whether a substitution affects protein function.
      1. SFT classifies substitutions as “tolerated” or “deleterious”.
    ii. CADD (15)—A tool for scoring the deleteriousness of a single nucleotide variants.
      1. C-scores range from 0 to 99. As the score increases, pathogenicity increases in a scaled manner.
- IntSplice (https://www.med.nagoya-u.ac.jp/neurogenetics/IntSplice2/) (16)—a tool that predicts a splicing consequence of a single nucleotide variation at intronic positions −50 to −3 close to the 3′ end of an intron in the human genome.
- TraP (https://trap-score.org/home) (17)—a tool constructed to evaluate a single nucleotide variant's ability to cause disease by damaging the final transcript. Can analyze exon as well as intron variants.
  a. Scores for exon and intron variants vary in range but are all classified by percentile. Scores above the 90th percentile (0.174 for intronic variants) are considered “possibly damaging” and those above the 97.5th percentile (0.289 for intronic variants) “probably damaging”.

The identified variants were then cross-referenced with two population databases using the gnomeAD browser (https://gnomad.broadinstitute.org/): ExAC and gnomAD (18). Identified variants were also queried in the ClinVar database for validation (19). Both the population databases and ClinVar were last accessed August 29, 2023.

To verify the accuracy of these web-based platforms and databases, we inputted 2 known Pathogenic variants from a different subject; c.2864 C > A (p.Ser955*) (nonsense) and c.10550-1 G > A (splice acceptor variant). These analysis platforms variants produced results that were consistent with current classification protocols.

The ACMG-AMP Guidelines provide a nomenclature for specific pieces of evidence support variant classification [see Table 4 in (8) for the complete list]. In this study, we cite the following categories of evidence in our analysis:

1. PP3: multiple lines of computational evidence support a deleterious effect on the gene or gene product (conservation, evolutionary, splicing impact, etc.)
2. PM2: absent from controls (or at an extremely low frequency if recessive) in Exome Sequencing Project, 1,000 genomes, or ExAC.
3. BP4: multiple lines of computational evidence suggest no impact on gene or gene product (conservation, evolutionary, splicing impact, etc)
4. BP5: variant found in a case with an alternate molecular basis for disease.

## 3 Results

These results are summarized in Table 2.

**Table 2:** Results of in-silico prediction analysis.

Subject	Variant		Results				Summary
	Inheritance	Description	ExAC/gnomAD allele counts	PhyloP	PolyPhen2/SIFT/MutTaster	CADD	
					TraP and IntSplice if applicable^a^		
1	Paternal	c.16172T > G(p.Leu5391Arg)	0/0	4.74	PD (1.000)/D/D	D (25.3)	Both Subject 1's variants are predicted deleterious
	Maternal	c.2035C > T(p.Arg679Trp)	2/3	3.398	PD (1.000)/D/D	D (35)	
2	Unknown	c.12286-10T > C(intronic)	5/14	–	TraP: 0.211 (93rd percentile, “Possibly Damaging”)^a^	N (8.516)	Both Subject 2's variants are predicted non-deleterious
					IntSplice: Normal		
	Unknown	c.1283A > G(p.Asn428Ser)	2/1	3.673	B (0.03)/N(0.244)/B	N (17.82)	

(–), Not available; D, deleterious or damaging; PD, probably damaging; N, neutral; B, benign. PolyPhen2 results indicate the interpretation and the score in parentheses. The CADD column indicates the interpretation C-score in parentheses.

^a^ The TraP score threshold for a label of “possibly damaging” is the 90th percentile of scores (0.174). There is a higher threshold for “probably damaging” at the 97.5th percentile (0.289). Thus, given that the score of the variant noted is in the 93rd percentile, we note this as weak evidence of any pathogenic effect and a natural outlier against our competing body of evidence.

### 3.1 Subject 1

Molecular testing identified 2 *ADGRV1* variants: Paternal c.16172 T > G (p.Leu5391Arg) and maternal c.2035 C > T (p.Arg679Trp). Both variants are missense and currently classified as VUS (Table 1).

At the time of testing, Invitae reported that algorithms developed to predict the effect of missense changes on protein structure and function are either unavailable or do not agree on the potential impact of these variants (as of 2021) citing SIFT and PolyPhen-2 results.

The paternal c.16172 T > G (p.Leu5391Arg) has a PhyloP score of 4.74, indicating the position is evolutionarily conserved. PolyPhen-2 predicted this variant to be “probably damaging” with a score of 1.000. SIFT predicted the variant as deleterious with a 79% accuracy. MutTaster predicted the variant as deleterious. The CADD score for this variant is 25.3 (deleterious) with an accuracy of 71% (PP3). This variant has not been reported in the population databases queried to date (PM2).

The maternal variant c.2035 C > T (p.Arg679Trp) has a PhyloP score of 3.398, indicating the position is evolutionarily conserved. PolyPhen-2 predicted this variant to be “probably damaging” with a score of 1.000. SIFT predicted the variant as deleterious with a 79% accuracy. MutTaster predicted the variant as deleterious. The CADD score for this variant is 35 (deleterious) with an accuracy of 84% (PP3). The allele frequency in the combined gnomAD and ExAC database is 0.00001220, allele count 3 (PM2). This variant has been reported in the African/African American, South Asian, and Bulgarian populations, but not our subject's ethnic subgroup.

### 3.2 Subject 2

Molecular testing identified 2 *ADGRV1* variants: c.12286-10 T > C, (intronic) and c.1283 A > G (p.Asn428Ser) (missense) (Table 1). Both variants are currently VUS. The subject's mother was negative for both variants, and the subject's father was unavailable for testing, thus the inheritance pattern could not be confirmed. There are three possibilities: (1) Both variants are in cis from the father, (2) The father is a carrier for one variant and the other arose *de novo*, (3) both variants arose *de novo* which is highly unlikely. Since we cannot eliminate any of these scenarios, these variants still require further investigation.

According to Invitae's entry into ClinVar, variant c.12286-10 T > C is located in intron 59 and does not directly change the amino acid sequence of the *ADGRV1* protein. It has not been reported in individuals with *ADGRV1*-related conditions. Invitae also states algorithms developed to predict the effect of sequence changes on RNA splicing suggest that this variant may disrupt the consensus splice site. IntSplice predicted the variant as normal. The CADD score generated for this variant was 8.516 (neutral) predicted with 76% accuracy (BP4). The TraP score was 0.211, which falls in the 93rd percentile approximately. The 93rd percentile is considered “possibly damaging”. This TraP value only slightly surpasses the “possibly damaging” threshold of the 90th percentile and is nowhere near the threshold for “probably damaging” (97.5th percentile). Thus, although this value TraP value may suggest pathogenicity, this value is an outlier compared to our body of evidence.

The allele frequency in the gnomAD browser is 0.00005236, allele count 14. The majority of the alleles detected were in the African American population (10/14), our subject's ethnic group. This variant was also detected in the East Asian and Latino/Admixed American populations.

Variant c.1283 A > G (p.Asn428Ser) has a PhyloP score of 3.673, indicating the position is evolutionarily conserved. PolyPhen-2 predicted the variant as benign with a score of 0.03. SIFT predicted the variant as neutral (0.244) with an accuracy of 81%. MutTaster predicted the variant as benign. The CADD score generated for this variant was 17.82 (neutral) with an accuracy of 56% (BP4). The allele frequency in the gnomeAD browser is 0.000004012, allele count 1. The singular allele reported was detected in the African American population, our subject's ethnic group.

During the course of this study, Invitae (San Francisco CA) reclassified both Subject 2's *ADGRV1* variants as Likely Benign and he was formally diagnosed with Harboyan Syndrome (BP5), which supports these findings.

## 4 Discussion

Usher Syndrome is the second most common cause of syndromic SNHL with USH2 representing two-thirds of all cases. Louisiana in particular has an increased prevalence of Usher Syndrome due to geographical isolation leading to founder populations (3). Early identification of causative variants via NGS and diagnosis is critical to providing families with anticipatory guidance and early intervention before symptoms progress (2–4). Early molecular diagnosis through NGS has important consequences for disease management including earlier access to eye exams, educational resources, cochlear implantation, and psychosocial impacts on families (1–4).

In this study, we identified 2 *ADGRV1* compound heterozygotes. Both of these subjects presented with mild-moderate bilateral SNHL and normal vestibular findings, consistent with the USH2 phenotype (4). Neither exhibited features of RP at this time likely due to their young age (2–4). Both of these subjects are carriers for multigenic VUS (Table 1). We chose to perform a preliminary analysis of these *ADGRV1* variants given their likelihood of producing a progressive disease due to compound heterozygous inheritance. Our data provides evidence of pathogenicity in Subject 1's *ADGRV1* variants, and our analysis supports the “Likely Benign” classification of Subject 2's *ADGRV1* variants (Table 2). Our analysis supports ongoing efforts to provide more information and ultimately reclassify VUS so clinicians may extract higher diagnostic yield from inconclusive genetic testing reports. We further aim to support clinicians in this endeavor by providing an algorithm for clinicians analyzing genetic reports with multiple variants (Figure 1). In the case of our subjects, Subject 1's results may warrant more aggressive screening measures for signs and symptoms of USH2. In contrast, Subject 2's analysis puts his likelihood of developing USH2 extremely low, and thus allows clinicians to focus on his *SLC4A11* variants as a more probable cause for his presentation.

The genes involved in USH1, 2, and 3 are collectively termed “Usher genes” (2–4). USH2 is distinct from other subtypes of Usher Syndrome (USH1 and USH3) based on the severity of SNHL and age of onset of RP; USH1 and USH3 are also genetically distinct from USH2. USH2 is linked to 3 causative genes: *USH2A, ADGRV1, and WHRN*. The gene *ADGRV1* (also known as *VLGR1*) produces a very large calcium binding G-coupled protein receptor expressed in the human ear, retina, and central nervous system. *ADGRV1, USH2A* and *WHRN* form the ankle link that connects neighboring stereocilia during hair cell development and induces cell spreading as the hair cells differentiate (3, 4, 6, 7). There is also evidence that *ADGRV1* is involved as a metabotropic mechanosensor during this process (6, 7). Absence of *ADGRV1* leads to misshapen outer hair cell bundles, accounting for the early high-frequency hearing loss. *ADGRV1* is known to participate in a “protein interactome” with Usher genes and many others (3, 4, 6, 7). The molecular mechanisms behind this interactome are not fully understood, however *ADGRV1* has been shown to participate in downstream signaling related to gene regulation, pre-RNA splicing, transcriptional control, vesicle docking/transport control, and balancing Ca^2+^ homeostasis within this interactome (4, 6, 7).

Variant analysis guidelines are set by the ACMG-AMP and include recommendations regarding the use of computational (*in-silico*) prediction programs and population databases, such as those utilized in this study. Many *in-silico* prediction programs are freely available to researchers via web-based platforms and provide preliminary results faster than other experimental methods. The algorithms employed in these programs consider the position of the variant (conservation throughout evolution), impact on the genomic transcript, and impact on protein structure in order to create a prediction. Other important considerations many algorithms incorporate include function of the protein product as well as variant type (4, 8). These platforms are best suited to analyze missense variants (single nucleotide substitutions), which represented 3 out 4 *ADGRV1* variants in our cohort (8). The other variant type included intronic variant located in a splice site. We utilized IntSplice and TraP for analysis of the splice site intronic variant (16, 17).

The ACMG-AMP guidelines also allow population data to inform its variant classification system. All of the *ADGRV1* variants detected in this study had a <1% frequency or were absent altogether in the population databases searched. If a variant is absent from population databases or, if it is a recessive variant, has an extremely low frequency in population databases, the ACMG-AMP considers this as moderate evidence of pathogenicity (PM2). As population studies continue to collect more data, some of these frequencies may rise and thus lead to a Benign or Likely Benign reclassification (8, 18). It is vital for physicians to understand the dynamic nature of these classification systems and how to use these results to counsel families appropriately (5).

The pace at which data is produced from NGS has never been faster or more affordable due to technological advances (2–5, 8). This is reflected by the increasing number of new variants classified as VUS and increasing likelihood of reclassification. Compound heterozygotes with VUS must be followed closely to ensure that delay is minimal between reclassification, familial counseling, and further treatment or testing if appropriate (2, 8). In terms of the bioinformatics analysis utilized in the present study, this method cannot be used for diagnostic purposes in a clinical setting, although it is highly useful for gaining information on novel variants and/or VUS (8). Physicians should build strong relationships with medical geneticists as they are most equipped to discuss the nuances of molecular testing with patients before and after diagnosis; however, other practitioners should understand how to interpret molecular results (1). Figure 1 demonstrates how physicians can analyze a genetic report and build a differential diagnosis based on these results. This process should be done with the guidance of medical geneticists.

### 4.1 Limitations and future directions

The study provides insights about the potential genetic drivers of pediatric sensorineural hearing loss. However, limitations must be acknowledged. In the present study, we use the targeted next generation sequencing Invitae Comprehensive Deafness Panel^©^ (San Francisco CA). Targeted sequencing panels are clinically approved genetic tests that allow the identification of genetic changes associated with health conditions of interest (e.g., hearing loss, breast cancer) but only on already known exon variants and adjacent intronic sequences. Future clinical and research applications include whole exome sequencing (WES) or whole genome sequencing (WGS) in unresolved cases after targeted panel testing followed by bioinformatics analysis. Peripheral blood or saliva samples would be obtained from patients following informed consent for DNA isolation and subsequent exome or genome sequencing. Due to the large datasets obtained from next generation sequencing, raw genetic data is typically stored on FASTQ files and converted to BAM files using tools implemented in the exome sequence data analysis pipelines (20, 21). Raw sequence is assembled, mapped and aligned to the reference genome GRCh38 (22), duplicate reads are removed using various tools (23, 24), and somatic mutations/variants (single nucleotide variants, i.e., SNVs and insertion/deletions, i.e., indels) are identified with various algorithms (22, 25–27). Variant calls that do not meet “PASS” criteria for these algorithms are removed, and only those passing at least two algorithms are retained, and validated by multiple separate algorithms (28–31). In the process of variant calling, pathogenicity is evaluated using many of the same algorithms utilized in the present study. Confirmatory molecular testing (e.g., RT-PCR, qPCR) allows for better understanding of genotype/phenotype correlations implicated in HL. Once validated, these newly identified genes and variants can then be added to the genome database for inclusion in future targeted hearing loss gene panel testing.

Additionally, the focus on single nucleotide variants (SNVs) limits the scope of analysis. It is conceivable that other genetic variants such as insertions and deletions (indels) and copy number variants (CNVs) are likely involved in hearing loss. In terms of the analysis itself, many of the programs utilized are only designed to analyze variants located in exons (coding regions of DNA) and not introns (non-coding regions). Although we applied two platforms that were able to analyze our intronic variant (IntSplice and TraP), predicting the pathogenicity of intronic variants is an active area of research since intronic variants can have deleterious effects on RNA splicing and thus protein function. MutTaster does have an intron function, there were no suitable transcripts available to analyze our intronic *ADGRV1* variant through this platform. In general, most algorithms for missense variant prediction are 65%–80% accurate when examining known disease variants. Many of these platforms also have low specificity, resulting in overprediction of missense changes as deleterious and are not as reliable at predicting missense variants with a milder effect (8). We utilized the PredictSNP platform for CADD and SIFT scores because it provides users with a measure of confidence in the accuracy of the prediction (13). In our study, all 4 variants analyzed fell within the standard accuracy range. Because many of these tools and algorithms have overlapping features, the ACMG-AMP considers multiple lines of *in-silico* prediction data as one piece of evidence for variant classification (8).

Another key limitation is the limited patient sample size. Although molecular testing is becoming more readily available in the management of HL, we are still encountering barriers to care (i.e., lack of insurance coverage, cost of genetic testing, stigmatization). There are likely many more variants and cases in the population that our study did not capture. Future directions on this front will include partnering with neighboring genetics departments to capture more subjects for analysis within our state and region.

Finally, this study focuses on genetics, but it is likely that the interactions among the genes and with the environment may be mediated by epigenetic factors. This limitation is particularly relevant to our cohort given that they have not exhibited the moderate-severe bilateral SNHL known of the USH2 phenotype. However, this difference could be due to lack of progression or the presence of other variants and epigenetic factors modifying the clinical presentation. Based on the interpretation by the clinical geneticist, it was unlikely that any of the other variants the subjects possessed would produce disease in isolation, however, these VUS may produce compounding effects that must be further studied (2, 4).

## 5 Conclusion

Our predictions for each variant studied were based on consistent results across *multiple in-silico* platforms, population data, and data available in ClinVar as well as the patient's medical record. We recommend that physicians build strong relationships with medical geneticists and carefully review their interpretation before making recommendations to families, particularly when addressing the VUS (5). Early molecular diagnosis through NGS is ideal, as families are then able to access a wide range of resources that will ultimately support the child as their condition progresses (1–4). Future studies must examine the effects of multigenic variants in hearing loss genes as they may collectively impact the phenotype. Further research using a large sample size and mapping other potential genetic and epigenetic determinants of hearing loss and the pathways they control is recommended. These will be the focus of future investigations.

## Data Availability

The original contributions presented in the study are included in the article/Supplementary Material, further inquiries can be directed to the corresponding authors.

## References

[1] Kılıç S Bouzaher MH Cohen MS Lieu JEC Kenna M Anne S. Comprehensive medical evaluation of pediatric bilateral sensorineural hearing loss. Laryngoscope Investig Otolaryngol. (2021) 6:1196–207. 10.1002/lio2.657 PMC8513426 34667865

[2] Bahena P Daftarian N Maroofian R Linares P Villalobos D Mirrahimi M Unraveling the genetic complexities of combined retinal dystrophy and hearing impairment. Hum Genet. (2022) 141:785–803. 10.1007/s00439-021-02303-1 34148116 PMC9035000

[3] Castiglione A Möller C. Usher syndrome. Audiol Res. (2022) 12:42–65. 10.3390/audiolres12010005 35076463 PMC8788290

[4] Delmaghani S El-Amraoui A. The genetic and phenotypic landscapes of Usher syndrome: from disease mechanisms to a new classification. Hum Genet. (2022) 141:709–35. 10.1007/s00439-022-02448-7 35353227 PMC9034986

[5] Pilarski R. How have multigene panels changed the clinical practice of genetic counseling and testing. J Natl Compr Canc Netw. (2021) 19:103–8. 10.6004/jnccn.2020.7674 33406496

[6] Knapp B Roedig J Roedig H Krzysko J Horn N Güler BE Affinity proteomics identifies interaction partners and defines novel insights into the function of the adhesion GPCR *VLGR1*/*ADGRV1*. Molecules. (2022) 27:3108. 10.3390/molecules27103108 35630584 PMC9146371

[7] Kusuluri DK Güler BE Knapp B Horn N Boldt K Ueffing M Adhesion G protein-coupled receptor VLGR1/ADGRV1 regulates cell spreading and migration by mechanosensing at focal adhesions. iScience. (2021) 24:102283. 10.1016/j.isci.2021.102283 33851099 PMC8024656

[8] Richards S Aziz N Bale S Bick D Das S Gastier-Foster J Standards and guidelines for the interpretation of sequence variants: a joint consensus recommendation of the American college of medical genetics and genomics and the association for molecular pathology. Genet Med. (2015) 17:405–24. 10.1038/gim.2015.30 25741868 PMC4544753

[9] Rim JH Lee JS Jung J Lee JH Lee S-T Choi JR Systematic evaluation of gene variants linked to hearing loss based on allele frequency threshold and filtering allele frequency. Sci Rep. (2019) 9:4583. 10.1038/s41598-019-41068-6 30872718 PMC6418148

[10] Katsonis P Wilhelm K Williams A Lichtarge O. Genome interpretation using in silico predictors of variant impact. Hum Genet. (2022) 141:1549–77. 10.1007/s00439-022-02457-6 35488922 PMC9055222

[11] Adzhubei IA Schmidt S Peshkin L Ramensky VE Gerasimova A Bork P A method and server for predicting damaging missense mutations. Nat Methods. (2010) 7:248–9. 10.1038/nmeth0410-248 20354512 PMC2855889

[12] Schwarz JM Cooper DN Schuelke M Seelow D. Mutationtaster2: mutation prediction for the deep-sequencing age. Nat Methods. (2014) 11:361–2. 10.1038/nmeth.2890 24681721

[13] Bendl J Musil M Štourač J Zendulka J Damborský J Brezovský J. PredictSNP2: a unified platform for accurately evaluating SNP effects by exploiting the different characteristics of variants in distinct genomic regions. PLoS Comput Biol. (2016) 12:e1004962. 10.1371/journal.pcbi.1004962 27224906 PMC4880439

[14] Ng PC Henikoff S. Predicting deleterious amino acid substitutions. Genome Res. (2001) 11:863–74. 10.1101/gr.176601 11337480 PMC311071

[15] Kircher M Witten DM Jain P O’Roak BJ Cooper GM Shendure J. A general framework for estimating the relative pathogenicity of human genetic variants. Nat Genet. (2014) 46:310–5. 10.1038/ng.2892 24487276 PMC3992975

[16] Takeda J-I Fukami S Tamura A Shibata A Ohno K. Intsplice2: prediction of the splicing effects of intronic single-nucleotide variants using LightGBM modeling. Front Genet. (2021) 12:701076. 10.3389/fgene.2021.701076 34349788 PMC8326971

[17] Gelfman S Wang Q McSweeney KM Ren Z La Carpia F Halvorsen M Annotating pathogenic non-coding variants in genic regions. Nat Commun. (2017) 8:236. 10.1038/s41467-017-00141-2 28794409 PMC5550444

[18] Karczewski KJ Francioli LC Tiao G Cummings BB Alföldi J Wang Q The mutational constraint spectrum quantified from variation in 141,456 humans. Nature. (2020) 581:434–43. 10.1038/s41586-020-2308-7 32461654 PMC7334197

[19] Landrum MJ Chitipiralla S Brown GR Chen C Gu B Hart J Clinvar: improvements to accessing data. Nucleic Acids Res. (2020) 48:D835–44. 10.1093/nar/gkz972 31777943 PMC6943040

[20] Wu J Mamidi TKK Zhang L Hicks C. Deconvolution of the genomic and epigenomic interaction landscape of triple-negative breast cancer. Cancers (Basel). (2019) 11:1692. 10.3390/cancers11111692 31683572 PMC6896043

[21] Wu J Mamidi TKK Zhang L Hicks C. Unraveling the genomic-epigenomic interaction landscape in triple negative and non-triple negative breast cancer. Cancers (Basel). (2020) 12:1559. 10.3390/cancers12061559 32545594 PMC7352267

[22] Zhang L Liu C Dong S. PipeMEM: a framework to speed up BWA-MEM in spark with low overhead. Genes (Basel). (2019) 10:886. 10.3390/genes10110886 31689965 PMC6896194

[23] Kairov U Molkenov A Sharip A Rakhimova S Seidualy M Rhie A Whole-genome sequencing and genomic variant analysis of Kazakh individuals. Front Genet. (2022) 13:902804. 10.3389/fgene.2022.902804 35899193 PMC9309552

[24] Pireddu L Leo S Zanetti G. SEAL: a distributed short read mapping and duplicate removal tool. Bioinformatics. (2011) 27:2159–60. 10.1093/bioinformatics/btr325 21697132 PMC3137215

[25] Rimmer A Phan H Mathieson I Iqbal Z Twigg SRF, WGS500 Consortium, Wilkie AOM Integrating mapping-, assembly- and haplotype-based approaches for calling variants in clinical sequencing applications. Nat Genet. (2014) 46:912–8. 10.1038/ng.3036 PMC4753679 25017105

[26] Cibulskis K Lawrence MS Carter SL Sivachenko A Jaffe D Sougnez C Sensitive detection of somatic point mutations in impure and heterogeneous cancer samples. Nat Biotechnol. (2013) 31:213–9. 10.1038/nbt.2514 23396013 PMC3833702

[27] Saunders CT Wong WSW Swamy S Becq J Murray LJ Cheetham RK. Strelka: accurate somatic small-variant calling from sequenced tumor-normal sample pairs. Bioinformatics. (2012) 28:1811–7. 10.1093/bioinformatics/bts271 22581179

[28] Ramos AH Lichtenstein L Gupta M Lawrence MS Pugh TJ Saksena G Oncotator: cancer variant annotation tool. Hum Mutat. (2015) 36:E2423–2429. 10.1002/humu.22771 25703262 PMC7350419

[29] 1000 Genomes Project Consortium, Auton A Brooks LD Durbin RM Garrison EP Kang HM Korbel JO A global reference for human genetic variation. Nature. (2015) 526:68–74. 10.1038/nature15393 26432245 PMC4750478

[30] Sherry ST Ward MH Kholodov M Baker J Phan L Smigielski EM dbSNP: the NCBI database of genetic variation. Nucleic Acids Res. (2001) 29:308–11. 10.1093/nar/29.1.308 11125122 PMC29783

[31] Tate JG Bamford S Jubb HC Sondka Z Beare DM Bindal N COSMIC: the catalogue of somatic mutations in cancer. Nucleic Acids Res. (2019) 47:D941–7. 10.1093/nar/gky1015 30371878 PMC6323903

